# Dietary Shift in a Barn Owl (*Tyto alba*) Population Following Partial Abandonment of Cultivated Fields (Central Apennine Hills, Italy)

**DOI:** 10.3390/ani14172562

**Published:** 2024-09-03

**Authors:** Gabriele Achille, Dan Gafta, Csaba Szabó, Fadia Canzian, Nazzareno Polini

**Affiliations:** 1 Laboratory of Protistology and Biology Education, University of Macerata, 62100 Macerata, Italy; g.achille@unimc.it; 2 Department of Taxonomy and Ecology, 3B Centre, Babeș-Bolyai University, 400015 Cluj-Napoca, Romania; 3 Department of Animal Nutrition and Physiology, Faculty of Agricultural and Food Sciences and Environmental Management, University of Debrecen, 4032 Debrecen, Hungary; 4 Studio Naturalistico PAN, 63900 Fermo, Italy; fadia.canzian@gmail.com (F.C.); npolini@gmail.com (N.P.)

**Keywords:** abandoned fields, barn owl, diet similarity, pellets, prey feeding guilds, prey taxon evenness, small mammals

## Abstract

**Simple Summary:**

Here, we aimed to detect differences in the composition, abundance distribution, and feeding group proportion of micromammal prey identified in barn owl pellets collected before (2004) and after (2012) the partial abandonment of cultivated fields within a cultural landscape in central Italy. The prey taxa were more evenly distributed in 2004 than in 2012, which translated into a change in the owls’ diet, basically an increased abundance of rodents (mice and rats) to the detriment of insectivores (shrews). This dietary shift is similar to that observed after agricultural intensification. These results offer a novel insight into the short-term effects of cessation of agricultural activities on small mammals and their predators, although third-party factors might also have been responsible for the observed dietary changes. If the present findings are further validated by more extensive research, they will constitute a scientific basis for controlling pests in fallow lands and still cultivated lands while ensuring the conservation of (near) threatened wildlife that is affected in one way or another by agricultural practices.

**Abstract:**

While most studies focused on the impact of intensive agriculture on the barn owl’s diet, little is known about the effect of cropland abandonment. We compared the taxon composition/evenness and feeding guild structure of small mammal prey identified in pellets collected before (2004) and after (2012) the abandonment of 9% of cultivated fields within a cultural landscape. Data on prey abundance per pellet were analysed through non-metric multidimensional scaling and permutational, paired tests. Prey taxon evenness in 2012 was significantly lower than in 2004. That induced a shift in prey taxon composition as indicated by the significantly lower dietary similarity compared with the random expectation. The increasing and declining abundance of *Murinae* and *Crocidurinae*, respectively, had the largest contribution to the differentiation of the diet spectrum. Insectivorous prey was significantly more abundant in 2004 compared to 2012, while the opposite was true for omnivorous prey. Our results suggest that even a small fraction of abandoned crops in the landscape might induce a detectable shift in the barn owl’s food niche. The dietary effects are similar to those observed after agricultural intensification, that is, an increase in the abundance of generalists to the detriment of specialist mammal prey.

## 1. Introduction

Agricultural land abandonment due to the progressive depopulation of rural areas is a growing trend in developed countries worldwide [1]. On the one hand, the cessation of agricultural activities represents a good opportunity to recover natural ecosystems, which is preferable in global warming. On the other hand, some short-term environmental issues related to land use abandonment exist, including biodiversity loss and increased frequency/intensity of fires [2]. Abandoned fields typically fall into two categories: former pastureland and ex-cultivated fields, both being initially colonised by weeds and/or thorny shrubs [3]. Such successional changes in vegetation also alter the composition and abundance of local fauna, as well as the predator-prey dynamics. For example, the abandoned fields can serve as refuge areas for small mammals during periods of disturbance [4,5] and can even modify the kinetics of rodent populations [6]. However, generalist species, like mice and rats, may be less affected since they can easily move across mosaic landscapes of alternating used and unused land [7]. Apart from one study revealing an increase in small mammal richness (but no monotonic response in their abundance) along a grassland (old-field-originated) chronosequence [8], no other research explicitly addresses the effects of crop abandonment on small mammal populations. Since any change in prey populations is eventually reflected in the upper trophic levels, a common study approach focuses on predators’ diets [9].

*Tyto alba*, a common and widespread nocturnal owl species, inhabits diverse environments, from lowlands to mountains, although it generally prefers warmer, lower-elevation areas [10,11,12]. The home range size of the barn owl varies significantly with landscape structure and prey availability, covering an area of about 1 to 25 km^2^ [13]. Barn owls notably tolerate human presence and are often found in anthropogenic environments [11,14]. Barn owls near human settlements have higher reproduction rates than those in more remote areas [15]. As generalists and opportunists, barn owls exhibit flexible behaviour, exploiting a variety of open habitats within agricultural landscapes [13].

The barn owl’s diet primarily consists of micromammals and feeds on insects, lizards, small birds, and bats [16,17]. Many authors reported that the foraging pattern, expressed as small mammal composition and taxonomic/trait diversity, was dependent on land use [18,19,20,21,22], agricultural intensification [23,24,25], and landscape structure [22,26]. It is currently acknowledged that the food niche breadth of the barn owl depends, among others, on the types and structures of existing habitats in cultural landscapes, reflecting the effects of habitat heterogeneity and agricultural activity on prey availability [20,21,27,28]. Intensive land cultivation impacts the barn owl’s diet, usually translating in a notable decline in specialist small mammals (i.e., insectivores), which are replaced by generalists (omnivores) like mice and rats [22,23,25,29] or, sometimes, by amphibians [30]. Thus, the barn owl’s feeding strategy is rather flexible since it can easily switch among different prey species when their availability changes [17,31]. These dietary changes are often related to biodiversity loss due to habitat fragmentation and homogenisation in cultural landscapes [32,33,34]. For this reason, the barn owl’s diet could indicate rodent abundance and diversity within its home range while playing an important role in biological rodent control [28,35,36].

This study explored how the barn owl’s diet spectrum changed following the partial abandonment of cultivated fields in the central Apennine foothills. To this end, we conducted analytical comparisons of repeated surveys on a single owl population, focusing on potential differences in (i) taxonomic richness, as well as dominance and composition of prey, and (ii) abundance of prey taxa/feeding guilds. We expected to detect sensible changes in all of these indicators but could not make reliable predictions about the direction of these changes.

## 2. Materials and Methods

### 2.1. Study Area

**Figure 1:**
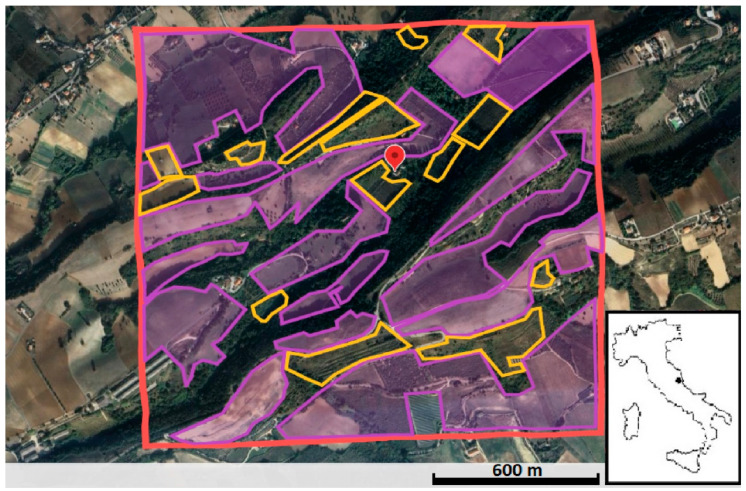
Map of the study area (red quadrat) showing the cultivated fields in 2004 (purple polygons) and the subsequently abandoned fields in 2012 (yellow polygons). The location of the owls’ roost, where the pellets were collected, is marked by the red balloon sign.

Given that habitat preferences may occur at a finer scale within the home range [37], a quadrat area of approximately 233 hectares surrounding the target site was delimited to evaluate changes in land use (Figure 1). In 2004, the cultivated fields covered approximately 55% of the area, while by 2012, the abandoned fields increased from zero to 9% at the expense of crops, and the wooded area remained unchanged (Table 1). The pellet collection site, located at N 43°05′59″ and E 13°46′23″, was centrally positioned within the defined study area (Figure 1).

Except for an expeditious survey that reported the occurrence of nesting birds in 13 out of 100 sampling plots distributed throughout the Marche Region [38], no scientific data is available regarding the density of barn owls in the study area.

The research area was located in a typical hilly landscape, with elevations not exceeding 600 m above sea level, within the western Adriatic watershed of the Marche Region, central Italy. Specifically, the study area was in Saltareccio, within the municipality of Lapedona, Fermo Province. The mean annual temperature is 16.2 °C, and the average annual precipitation is about 765 mm [39]. The landscape is predominantly agricultural, interspersed with narrow riparian groves and small woodlots. The dominant tree species in the potential zonal vegetation is the downy oak (*Quercus pubescens*). The hydrography features small reservoirs for field irrigation and primarily seasonal water bodies. Geologically, the area is characterised by arenaceous lithofacies, with occasional clay outcrops and minimal lithic presence [40].

The cultivated fields in the area are subject to crop rotation through monocultures of *Helianthus annuus*, *Sorghum vulgare*, *Medicago sativa*, *Zea mays*, or *Hordeum vulgare*. In addition to agricultural fields, the presence of olive tree orchards for domestic oil and olive consumption is worth noting.

The collection sites consisted of a masonry complex, including several rooms. Wads were collected from the ground in the spots frequently used by the barn owls. Particular attention was paid to the nest shelter, which was located in a double-roofed (loft-like) attic area open on one side. The nest area appeared very safe from predators, as it was only accessible to flying wildlife. The nest area had various abandoned debris, such as an old water collection tank. The latter seemed to have been a *Tyto alba* nest in recent years.

The area surrounding the collection site is heavily used for agricultural cultivation. The settlements are mostly isolated rural dwellings, with a few small towns lying in the upper part of the hills. The study area has five individual dwellings and a few medium-sized cattle/sheep sheds. The main disturbance factors that may affect the barn owls are attributable to the fast-moving car traffic along the provincial road, although it runs 4.5 km away from the collection site and is used by a relatively small number of vehicles. There is also a photovoltaic power station in the southern part of the study area.

### 2.2. Data Collection and Transformation

The analysis of barn owl pellets, which contain 70% to 100% of the preyed species [41], is a valuable method for examining variations in their trophic spectrum in response to environmental changes. Barn owl (*Tyto alba*) pellets were collected in the spring of 2004 and 2012. Due to their low availability in the field, the number of pellets collected in 2012 was much lower than that collected in 2004 (159 versus 535). Glue’s [42] method was employed for bone extraction, using sodium hydroxide solution or hydrogen peroxide to dissolve the fur and feather matrix. The prey mammals, reptiles, and birds were identified to the lowest possible taxonomical level by examining skulls, mandibles, teeth, bills, feet, and pelvises following the keys provided by [43,44,45,46,47]. Because several prey mammals could not be identified at least to the genus level, we grouped all items at the subfamily level before their input in numerical analyses. This way, the bias determined by the undesirable taxonomic nestedness (i.e., the presence of both lower and hierarchical upper-ranked taxa) in compositional data was removed at the expense of a lower taxonomic resolution. No data on inter-annual changes in the abundance of prey taxa were available.

The prey taxa (exclusively micromammals) were classified into three feeding guilds: insectivores, herbivores, and omnivores. The observed prey reptiles and birds were not considered in this study, as they were represented by very few individuals and taxa in the collected pellets. In addition, both reptiles and birds are not usually part of the barn owl’s diet [17,27].

### 2.3. Numerical Data Analysis

In all analyses, we treated the empirical data from 2004 and 2012 as paired since they originated from pellets collected during repeated surveys in the same area. Due to the different sample sizes in the paired datasets, all analyses were performed on densities of either prey taxa or prey-feeding guilds rather than individual counts. Therefore, for each taxon/guild, the number of target individuals was divided by the total number of pellets recorded in the given survey, and the result is hereinafter referred to as abundance.

The ordination of prey taxa in the bidimensional space determined by the two surveys was performed through nonmetric multidimensional scaling (NMDS) applied to the matrix of Euclidean distances. The purpose was to discern the most discriminant (distant) taxa, namely those that contributed most to the dietary change between 2004 and 2012.

Prey taxon evenness was assessed using Pielou’s J index, which equally weights common and rare taxa [48]. The magnitude of dietary shift between 2004 and 2012 was evaluated using the Petraitis [49] index of niche overlap, with values ranging from 0 (no overlap or complete separation) to 1 (perfect overlap or similarity). The significance of the difference in prey taxon diversity and evenness, and in the abundance of prey-feeding guilds between 2004 and 2012, was estimated separately using a permutational, paired, two-sided test under the null hypothesis of equal observed and simulated differences. The significance of diet similarity between the two paired surveys was estimated using a permutational, left-sided test under the null hypothesis of equal or greater niche overlap than the random expectation. These permutational tests were performed using a quantitative shuffle and swap algorithm, randomly reassigned prey individuals among taxa 9999 times while keeping the original total number of prey individuals/taxa fixed. Such a null model retained the observed niche breadth of the owls and the number of unexploited resource categories in each year but randomly altered which particular resource categories were used [50]. Since all null distributions of the target statistics were skewed, a non-parametric procedure was employed to calculate the associated standardised effect sizes and adjusted *p*-values [51]. Values outside the two-sigma interval (−1.96, +1.96) were considered statistically significant at the 5% alpha probability level.

All statistical analyses were performed in the R environment using specific packages: ‘vegan’ [52], ‘coin’ [53], and ‘spaa’ [54].

## 3. Results

**Figure 2:**
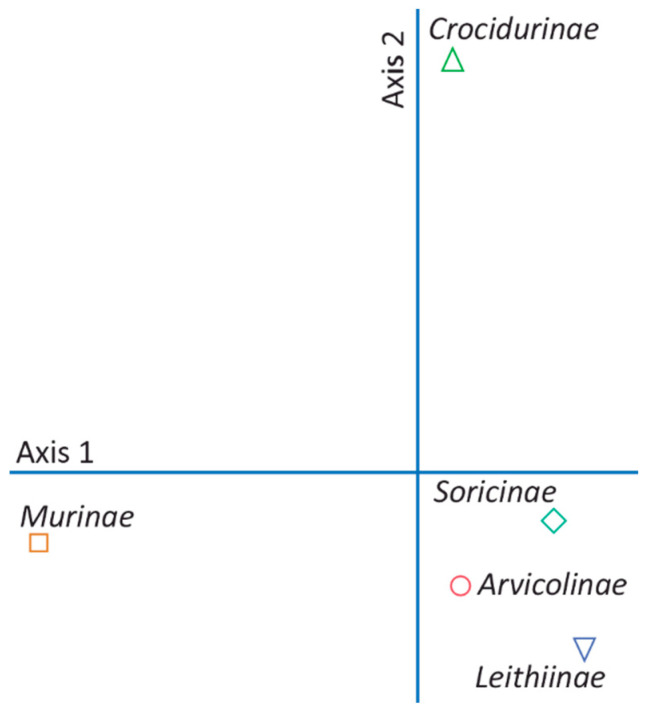
Ordination of prey taxa based on their abundance in pellets collected in 2004 and 2012 (NMDS final stress = 8.476 × 10^−6^).

The observed difference in prey taxon evenness between 2012 and 2004 was negative and significantly larger in absolute value than its simulated counterpart (Table 2). The dietary similarity (niche overlap) between the two survey years was significantly lower than expected under the null model (Table 2). The *Murinae* and *Crocidurinae* were the most important prey taxa in terms of their contribution to the differentiation of the diet spectrum between 2004 and 2012 (Figure 2). The abundance of the *Murinae* and *Crocidurinae* individuals increased by 8.8% and decreased by 24.9% after the partial abandonment of croplands. The dominant species in the two groups were *Apodemus sylvaticus* and *Mus musculus*, and respectively, the aggregate of *Crocidura* species and *Suncus etruscus*.

**Figure 3:**
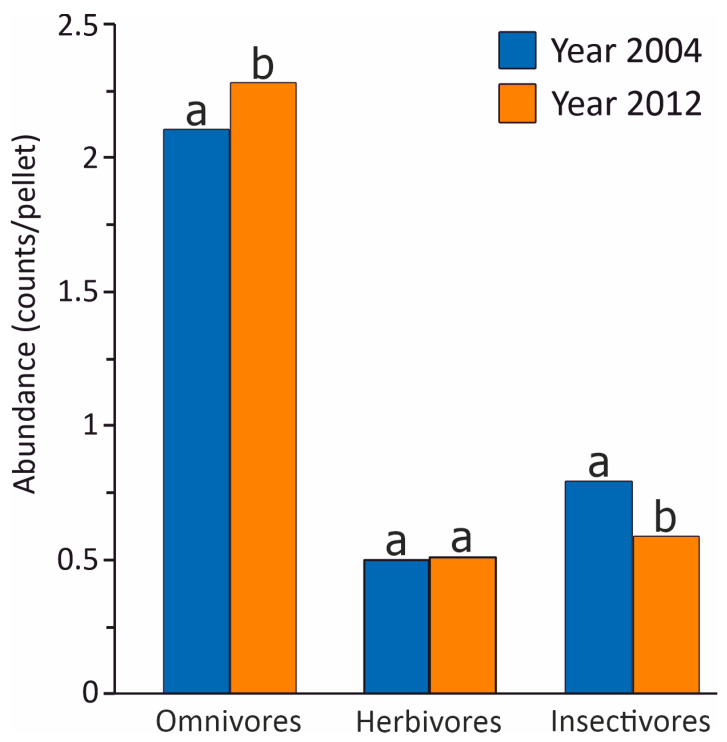
Abundance distribution of prey-feeding guilds in the years 2004 and 2012. Pairs of the same and different letters refer to non-significant and, respectively, significant differences between the two surveys.

The abundance of insectivorous prey was significantly higher in 2004 compared to 2012, while the omnivorous prey was significantly less abundant in 2004 than in 2012 (Table 2 and Figure 3). However, no significant differences were found between the paired abundances of herbivorous prey (Table 2 and Figure 3).

## 4. Discussion

After the partial abandonment of croplands, an increased dominance among prey taxa was observed, which translated into a weak but significant shift in the barn owl’s foraging niche. Such an adjustment might be related to changes in both habitat characteristics and prey availability within the barn owls’ hunting range [11]. In particular, the observed dietary shift was mainly due to an increase in the abundance of omnivorous prey (especially *Murinae*) to the detriment of insectivorous prey (especially *Crocidurinae* and, to a lesser extent, *Soricinae*). Besides, *Apodemus sylvaticus* (the most abundant *Murinae* species in our samples) was observed elsewhere to respond positively to the decrease in cropland area and the increase in fallow lands within cultural landscapes [24,55,56]. On the other side, the reduction in the abundance of *Crocidura* sp. and *Suncus etruscus* (the best-represented *Crocidurinae* in our samples) may be related to the negative effects of weed vegetation development and bush encroachment, the latter acting at much larger scales than our study area [19,57,58,59]. The apparent positive relationship between *Crocidura* species abundance and the proportion of croplands revealed in our study contradicts the opposite relationship observed by Horváth et al. [60] but agrees with other reports [61,62,63]. The dense and relatively tall weed/scrub vegetation developed in abandoned old fields is likely to lower the efficiency of owls’ predation on small mammals, suggesting that prey accessibility may be more important than prey density in the choice of foraging habitats [17,19,31,64,65]. Although fine-scale variations in capture success and habitat preference depend on factors other than prey availability [66], the barn owl is considered a good sampler of small mammal communities in open habitats [17,67,68]. Interestingly, the pattern implying the increase/decrease of omnivorous/insectivorous prey is similar to that often observed after agricultural intensification [22,25,29,69], which—in terms of anthropogenic pressure—represents exactly the opposite of cropland abandonment. The decline in the consumption of small insectivorous mammals is actually generalised in Europe [70]. Moreover, in accordance with the ongoing tendency of generalist-for-specialist prey species replacement [19,23,25,71,72,73], we noticed an increase in the abundance of synanthropic mice and rats (*Murinae*). All these confirm that small mammals can closely track land-use changes at a landscape scale [19,74], but not necessarily the direction of these changes, at least in the short term. However, the dominance of omnivorous prey should eventually decline in time to the benefit of insectivores as abandoned croplands are covered by mid-late successional vegetation.

The low number of pellets found in 2012 was probably the consequence of a decline in the size of the barn owl population after the partial abandonment of the croplands. This hypothesis finds some support in the negative effect of temporal instability of agricultural habitats and reduced structural diversity in cultural landscapes on barn owl reproductive success [65,75]. The presumed reduction in the barn owl population size is actually in line with the general declining trend reported across Europe [19,25,76,77,78] but may have been in part determined by local stochastic factors, e.g., parasite/disease outbreaks or extreme weather conditions.

All these results suggest that the small fraction of abandoned crops (about 9%) within the barn owl’s hunting range area might be enough to produce detectable changes in the dietary spectrum of barn owls. Several other studies reported the ability of barn owls to switch or widen their feeding niche by hunting many potential alternative prey according to land use changes in cultural landscapes [19,20,28,30,79].

The present study has some inexorable limitations due to some particular conditions that could not be controlled, of which the most important were the low pellet availability in the follow-up survey and the low taxonomic resolution reached in the identification of several prey remains. According to Contoli [80], the minimum number of prey needed to analyse the trophic spectrum of *Tyto alba* should not be less than 175 units, while Purger and Szép [81] inferred a minimum of about 300 pellets for the same purpose. As a consequence, the number of prey taxa detected in 2012 was probably underestimated. For this reason, but also because a series of prey individuals could not be identified, at least to the genus level, we could not properly estimate the difference in prey richness between the two paired surveys. Finally, we cannot exclude the possibility that third-party factors (e.g., year-to-year oscillation in shrew and rodent populations or excessive local use of pesticides and fertilisers) may have determined the observed dietary shift.

## 5. Conclusions

Overall, our results suggest that even a small fraction (like 9% in the present case) of abandoned crops in the landscape confined to their home range might induce a sensible shift in the barn owl’s food sources. This is most likely determined by changes in the availability of different prey whose population size depends directly (e.g., granivores) or indirectly (e.g., insectivores) on the extent of cultivated land. The novel finding of the present study is that the effect of cropland abandonment on the diet of barn owls is similar to that observed after agricultural intensification, that is, an increase in omnivorous prey to the detriment of their insectivorous counterparts. Since our results are ascribed to a single owl population and a particular landscape configuration, further research is needed to validate and perhaps generalise the patterns disclosed herein.

## Figures and Tables

**Table 1:** The area covered by different land-use categories within the study territory in 2004 and 2012.

	Area (ha)	
Survey Years	2004	2012
Cultivated fields	128.78	107.92
Abandoned fields	0	20.86
Scrub	41.97	41.97
Woods	52.01	52.01
Human built-up areas	10.30	10.30

**Table 2:** Significance and effect size of paired differences (2012–2004) in terms of prey taxon evenness, owls’ food niche breadth, and abundance of prey feeding guilds.

Dietary Feature	Observed Difference	Simulated Mean Difference	Standardised Effect Size	*p*
Prey taxon evenness	−0.06935	−0.00163	−3.891	0.0001
Food niche overlap	0.98656	0.99523	−1.996	0.0459
Omnivorous prey	0.17206	−0.01143	2.228	0.0259
Herbivorous prey	0.01224	−0.01177	0.295	0.7683
Insectivorous prey	−0.20762	−0.01130	−2.354	0.0186

## Data Availability

The raw data used in the study are included in the Supplementary Material (S1).

## Supplementary Materials

The following supporting information can be downloaded at https://www.mdpi.com/article/10.3390/ani14172562/s1; the raw data used in the study are included in the supplementary material (S1).
